# Whole-Genome Sequence and Interaction Analysis in the Production of Six Enzymes From the Three *Bacillus* Strains Present in a Commercial Direct-Fed Microbial (Norum™) Using a Bliss Independence Test

**DOI:** 10.3389/fvets.2022.784387

**Published:** 2022-02-22

**Authors:** Daniel Hernandez-Patlan, Bruno Solis-Cruz, Juan D. Latorre, Ruben Merino-Guzman, Miguel Morales Rodríguez, Catie Ausland, Xochitl Hernandez-Velasco, Oscar Ortiz Holguin, Ramiro Delgado, Billy M. Hargis, Pallavi Singh, Guillermo Tellez-Isaias

**Affiliations:** ^1^Laboratorio 5: LEDEFAR, Unidad de Investigacion Multidisciplinaria, Facultad de Estudios Superiores (FES) Cuautitlan, Universidad Nacional Autonoma de Mexico, Cuautitlán Izcalli, Mexico; ^2^Division de Ingeniería en Nanotecnología, Universidad Politécnica del Valle de Mexico, Tultitlán, Mexico; ^3^Department of Poultry Science, University of Arkansas, Fayetteville, AR, United States; ^4^Departamento de Medicina y Zootecnia de Aves, Facultad de Medicina Veterinaria y Zootecnia, Universidad Nacional Autonoma de Mexico (UNAM), Mexico City, Mexico; ^5^Department of Biological Sciences, Northern Illinois University, DeKalb, IL, United States; ^6^Biodigest S.A.S., Cali, Colombia; ^7^Nutriavícola S.A., Cali, Colombia

**Keywords:** direct-fed microbial, genome sequence, enzymes, bliss analysis, *Bacillus*

## Abstract

The three *Bacillus* strains present in Norum™ were initially selected by their excellent to good relative enzyme activity (REA) production score for amylase, protease, lipase, phytase, cellulase, β-glucanase, and xylanase. Further studies confirmed that the three isolates also showed an antibacterial activity, Gram-positive and Gram-negative poultry pathogens. Norum™ (Eco-Bio/Euxxis Bioscience LLC) is a *Bacillus* spore direct-fed microbial (DFM). The *Bacillus* isolates were screened and selected based on *in vitro* enzyme production profiles. Moreover, in chickens fed high non-starch polysaccharides, this DFM demonstrated to reduce digesta viscosity, bacterial translocation, increase performance, bone mineralization, and balance the intestinal microbiota. In the present study, we present the whole-genome sequence of each of the three isolates in Norum™, as well as the synergistic, additive, or antagonistic effects on the enzyme production behavior of the three *Bacillus* strains and their combinations when grown together vs. when grown individually. The whole-genome sequence identified isolate AM1002 as *Bacillus subtilis* (isolate 1), isolate AM0938 as *Bacillus amyloliquefaciens* (isolate 2), and isolate JD17 as *Bacillus licheniformis* (isolate 3). The three *Bacillus* isolates used in the present study produce different enzymes (xylanase, cellulase, phytase, lipase, protease, and β-glucanase). However, this production was modified when two or more *Bacillus* strains were combined, suggesting possible synergistic, antagonistic, or additive interactions. The Bliss analysis suggested (*p* < 0.05) that the combination of *Bacillus* strains 1–2 and 1–2–3 had intermediate effects and predicted that the combination of *Bacillus* strains 2–3 could have better effects than the combination of all the three *Bacillus* strains. In summary, the current study demonstrated the need of selecting *Bacillus* strains based on quantitative enzyme determination and data analysis to assess the impacts of combinations to avoid antagonistic interactions that could limit treatment efficacy. These results suggest that using *Bacillus* strains 2–3 together could lead to a new generation of DFMs with effects superior to those already examined in *Bacillus* strains 1–2–3 and, therefore, a potential alternative to growth-promoting antibiotics. More research utilizing poultry models is being considered to confirm and expand the existing findings.

## Introduction

Recent regulations to avoid antibiotics in animal production have led to the evaluation of new viable alternatives in terms of efficacy, costs, acceptability, and practicability (1). Despite the numerous alternatives that have been evaluated to replace antibiotics in the modern poultry industry, the group of probiotics has shown promising results (2). Despite the success of the lactic acid bacteria (LAB) probiotic for use in commercial poultry, there is still a pressing need for shelf-stable, cost-effective, and feed-stable (tolerance to the heat pelletization process) commercial probiotics to increase compliance and widespread use (3).

Bacterial spore formers, mainly of the genus *Bacillus*, are among the many probiotic products available today. Some (but not all) have been shown to prevent certain gastrointestinal disorders when used in their spore form, and the diversity of species used, and their applications are astounding. While not all *Bacillus* spores are heat tolerant, some isolates are the most strenuous life forms on the planet and can survive in scorching environments (4–6). Several studies conducted in our laboratory have shown that antimicrobial substances can be released by live vegetative cells or endospores against Gram-positive bacteria like *Clostridium perfringens* and *Clostridium difficile* (7, 8), as well as food-borne pathogens like *Salmonella* Enteritidis (9). As a result of these findings, products containing *Bacillus* spores are sold as probiotics. They have potential advantages over more common LAB products because they can be used as direct-fed microbials (DFM). Studies in our laboratory showed that DFM could ameliorate the severity of aflatoxicosis (10, 11) and necrotic enteritis in broiler chickens (12). More recently, we have shown that the *Bacillus* spores used as DFM improved intestinal integrity, bone mineralization, and reduced ammonia excretion in turkey poults fed with a rye-based diet (13). Interestingly, some, but not all, isolates of ingested *Bacillus* spores have been shown to germinate in the small intestine. According to scientific evidence, spores are just not passing through the gut; they have a close relationship with the host cells and microflora, which can enhance their probiotic potential (14). The sporulated form of these microorganisms can germinate in the aggressive conditions of the gastrointestinal tract and produce biofilms to protect themselves and tolerate the acidic pH, high osmotic concentrations of sodium chloride, and bile salts (15).

Spores of *Bacillus* strains have shown to have a significant effect on improving growth, feeding efficiency, and the immune system due to maintaining balance in the intestinal ecosystem and reducing emission of ammonia in the excreta of broilers (16). Probiotic effects of these microorganisms have been associated with a considerable number of combined mechanisms, such as the competitive exclusion of common poultry pathogens, release of active antimicrobial substances against Gram-positive bacteria, improvement of intestinal morphology, immunomodulation, and the reduction of toxic compounds (17, 18). Besides these essential mechanisms, the use of *Bacillus* strains as feed probiotics in poultry production is related to their capacity to produce beneficial chemical compounds for the host such as bacteriocins, organic acids such as lactic acid, hydrogen peroxide, diacetyl, and carbon dioxide, as well as the production of extracellular enzymes, such as protease, lipase, cellulase, xylanase, phytase, and keratinase, which can improve digestion by breaking down some chemical bonds, increase nutrient absorption, decompose antinutrient agents in feedstuffs, and support the host endogenous enzymes (7, 19, 20).

There are some evidence to suggest that different *Bacillus* species and subspecies interact with the digesta and the gut microbiota in a variety of ways (16, 21, 22). A commercial DFM, containing two or more defined strains geared to boost poultry performance, must be selected to observe synergistic or additive effects to avoid compromising the efficiency of the product.

Norum™ (Eco-Bio/Euxxis Bioscience LLC, Fayetteville, AR, USA) is a *Bacillus* spore DFM, previously identified by 16S rRNA sequencing as *Bacillus amyloliquefaciens* (two isolates) and one isolate of *Bacillus subtilis*, which were isolated in our laboratory and screened based on *in vitro* enzyme production profiles and pathogen reduction (7–9, 12). In addition, these isolates were shown to reduce digesta viscosity, bacterial translocation, improve performance, bone quality parameters, and balance intestinal microbiota in chickens raised with rye-based diets or corn distiller-dried grains with solubles (23, 24). Hence, the objectives of the present study were to complete the genome sequence of each of the three isolates in Norum™ and to evaluate the synergistic, additive, or antagonistic effects on the enzyme production behavior of the three *Bacillus* strains and their combinations by the Bliss independence analysis. Therefore, evaluating when they are grown together in comparison with when they are individually cultivated is vital for the proper selection of suitable probiotic strains with the potential as a commercial probiotic for poultry.

## Materials and Methods

### *Bacillus-*Direct Fed Microbial

Norum™ is a commercial *Bacillus*-direct fed microbial patented by the University of Arkansas (25). The three *Bacillus* strains present in Norum™ were initially selected by their excellent to good relative enzyme activity (REA) production score for amylase, protease, lipase, phytase, cellulase, β-glucanase, and xylanase (7, 8). Further studies confirmed that the three isolates also showed an antibacterial activity against Gram-positive and Gram-negative poultry pathogens (8, 9).

### Genome Sequence

Previous identification using 16S rDNA sequence revealed that the isolates of the commercial DFM (Norum™) were one *Bacillus subtilis* isolate (isolate 1) and two *Bacillus amyloliquefaciens* (isolates 2 and 3) (7). From each isolate, DNA extraction from overnight cultures was performed with a DNeasy UltraClean microbial kit (Qiagen LLC, Germantown, MD, USA) following the protocol of the manufacturer. Library preparation using Nextera XT DNA library preparation kit (Illumina, Inc., San Diego, CA, USA) and sequencing was performed at the University of Illinois at Chicago Sequencing Core (UICSQC) using a NextSeq 500 instrument (Illumina, Inc.) with 150-bp paired-end sequencing. Trimming was performed in the software package CLC Genomics Workbench v11.0.1 (Qiagen). Trimming was performed using default parameters with a threshold of Q20. Sequences demultiplexed in the BaseSpace cloud computing environment provided by the UICSQC. Genome assembly quality was determined by the QUAST quality assessment tool (26).

### Bacillus Strains and Culture Conditions

*Bacillus* isolates were independently grown in tryptic soy broth (TSB, Becton Dickinson, Sparks, MD, USA) at 37°C for 24 h. Then 100 μl of the culture of each of the *Bacillus* isolates were added to the selective culture media to produce enzymes as describe below.

#### Cellulase Production

For evaluation of cellulase activity, the cellulose-Congo red was used and consisted of 0.50 g of K_2_HPO_4_ (Fisher Scientific, San Francisco, CA, USA), 0.25 g of MgSO_4_ (Sigma Chemical Co, St. Louis, MO, USA), 1.88 g of ashed, acid-washed cellulose powder (J. T. Baker Chemical Inc., Phillipsburg, NJ, USA), 0.20 g of Congo red (J. T. Baker Chemical Inc., Phillipsburg, NJ, USA), and 1,000 ml of distilled water (27). Isolates were incubated at 37°C for 24 h.

#### Xylanase Production

For evaluation of xylanase activity, the medium used to screen *Bacillus* isolates contained 3 g of NaNO_3_, 0.5 g of K_2_HPO_4_, 0.2 g of MgSO_4_·7H2O, 0.02 g of MnSO_4_·H2O, 0.02 g of FeSO_4_·H_2_O, 0.02 g of CaCl_2_·2H_2_O, and 1,000 ml of distilled water. Besides, 1 g of yeast extract and 5 g of beechwood xylan (Sigma Chemical Co., St. Louis, MO, USA) were used as carbon sources (28). Isolates were incubated at 37°C for 24 h.

#### Phytase Production

For determination of phytase activity, *Bacillus* isolates were screened in a medium that contained 10 g of dextrose, 0.3 g (NH_4_)_2_SO_4_, 0.5 g of MgSO_4_, 0.1 g of CaCl_2_, 0.01 g of MnSO_4_, 0.01 g of FeSO_4_, 5 g of Na-phytate, and 1,000 ml of distilled water. The phytate media was autoclaved at 121°C for 15 min. Isolates were inoculated and incubated at 37°C for a maximum of 120 h (29, 30).

#### Protease Production

For evaluation of protease activity, a skim milk medium was prepared containing 25 g of skim milk, and 1,000 ml of distilled water. The mixture was stirred thoroughly and autoclaved at 121°C for 15 min. *Bacillus* strain was incubated at 37°C for 24 h (31).

#### Lipase Production

Lipase activity was assessed using the Spirit blue medium (Difco Laboratories, Detroit, MI, USA) composed of 10 g of pancreatic digest of casein, 5 g of yeast extract, 0.15 g of the dye spirit blue and 1,000 ml of distilled water. The medium was sterilized at 121°C for 15 min and cooled to 50°C in a water bath, before being mixed with 30 ml of a lipoidal solution prepared with 100 ml of olive oil, 1 ml of polysorbate 80, and 400 ml of warm water (60°C). Isolates were incubated at 37°C for 24 h (32).

#### β-Glucanase Production

This is composed of carboxymethylcellulose 0.5% (w/v) as sole carbon source and Mandels medium as nitrogen full complex (33, 34). The components were mixed, sterilized, and autoclaved at 121°C and 1 atm for 15 min. Isolates were incubated at 37°C for 24 h.

### Quantitative Assays for Enzyme Determination

For evaluating enzyme production in combinations of two or three isolates of *Bacillus*, equal volumes of each inoculum of *Bacillus* were added to the different culture media to have a final volume of 100 μl (50 μl of each inoculum for the combination of two strains and 33 μl of each inoculum for the combination of three strains). The determination of each enzyme was performed five times in independent *Bacillus* cultures (*n* = 5). The three *Bacillus* isolates used in the present study produce different enzymes (xylanase, cellulase, phytase, lipase, protease, and β-glucanase). Quantification of each enzyme was performed using commercial enzyme-linked immunosorbent assays kits as describe below.

#### Xylanase Production

With slight modifications, xylanase production was determined using a commercial assay kit (Megazyme International Ireland, Item No. XYLS 05/17, Bray, Co. Wicklow, Ireland). Briefly, 300 μl of the culture medium of each *Bacillus* isolate was placed in 1.5-ml Eppendorf tubes, followed by the addition of 300 μl of 0.1 N acetic acid solution. Subsequently, 8 μl of distilled water or 8 μl of control xylanase solution was added to each tube. Tubes were kept at room temperature for 20 min with occasional shaking and centrifuged at 1,500 × *g* for 10 min. Then 200 μl of each supernatant was placed in an Eppendorf tube containing a Xylazyme AX tablet. These tubes were incubated for 30 min at 50°C, and 800 μl of Trizma (2%, pH = 9.17) was added to each one to be later centrifuged for 10 min at 1,500 × *g*. Finally, 100 μl of the supernatants was placed in 96-well Bacti flat-bottom plates for measurement at 590 nm using an ELISA plate reader (Synergy HT, multimode microplate reader, BioTek Instruments, Inc., Winooski, VT, USA). Xylanase quantification was performed by interpolation on a calibration curve that was prepared under the same conditions as the samples.

#### Cellulase Production

The Azo-Barley Glucan method provided for a commercial kit with slight modifications (Megazyme International Ireland, Item No. K-MBGL 03/11, Bray, Co. Wicklow, Ireland) was followed for the quantification of cellulase. Briefly, 25 μl of CellG5 substrate solution was dispensed in 96-well plates and preincubated at 40°C for 3 min. Subsequently, 25 μl of the culture medium of each *Bacillus* isolate or a standard solution, previously preincubated at 40°C for 3 min, were placed in each well. At the end of the 30 min incubation period, 100 μl of Stopping Reagent were added. Absorbance was measured at 405 nm using an ELISA plate reader. For each set of assays, a reagent blank value was determined using an uninoculated culture medium, as well as a calibration curve.

#### Phytase Production

For phytase determination, a Megazyme kit (Megazyme International Ireland, Item No. K- PHYT 05/19, Bray, Co. Wicklow, Ireland) was used to make some modifications to the protocol. Briefly, protocol modifications consisted of reducing sample and reagent volumes by half. However, the main modification was in the preparation of the calibration curve, keeping the same temperature conditions and incubation times constant. The absorbance of samples, blanks, and calibration curve was measured on an ELISA plate reader set to 655 nm.

#### Lipase Production

Quantification of lipase was performed following the protocol of a commercial assay kit (Sigma-Aldrich, Item No. MAK047, MO, USA) with some modifications considering liquid samples. In this case, aliquots of 100 μl of each culture medium from the *Bacillus* isolates or only culture medium as blank were placed in 0.5-ml Eppendorf tubes containing 100 μl of a lipase assay buffer. The samples and blank were centrifuged at 13,000 × *g* for 10 min, and 50 μl of each supernatant was placed in 96-well plates. Subsequently, 50 μl of a master reaction mix containing 2% of lipase substrate was added to each well, and plates were incubated at 37°C for 3 min protected from light. In addition to the samples and blank, a positive control was prepared using a solution of known lipase concentration and to generate a calibration curve to quantify the samples by interpolation. Samples were measured at 450 nm using an ELISA plate reader after 3 min of incubation, followed by every 20 min, until one of the samples presented an absorbance of a similar magnitude to the last level of the calibration curve.

#### Protease Production

Protease activity was determined with a commercial kit (Thermo Scientific, Item No. 23263, IL, USA) using succinylated casein and trinitrobenzene sulfonic acid (TNBSA) as primary reagents. However, sample and reagent volumes and incubation times were modified and standardized for our purposes. Briefly, 100 μl of a succinylated casein solution or assay buffer was placed in two independent sets of microplate wells. Then 50 μl of the culture medium of each *Bacillus* isolate diluted 10 × or standards of a calibration curve was added to both sets of microplate wells, together with their respective blank, which contained all the elements except the succinylated casein solution. Plates were incubated for 60 min at room temperature. Finally, 50 μl of TNBSA working solution was added to each well and incubated for another 60 min. Absorbance of wells was measured on an ELISA plate reader set to 450 nm.

#### β-Glucanase Production

The β-glucanase production of each *Bacillus* isolate and their combinations was determined using a commercial assay kit (Megazyme International Ireland, Item No. K-MBGL 03/11, Bray, Co. Wicklow, Ireland) following the Azo-Barley glucan protocol with slight modifications. Briefly, 250 μl of the culture medium of each *Bacillus* isolate was placed in 1.5-ml Eppendorf tubes and 250 μl of an extractant buffer solution (40 mM sodium acetate buffer with 40 mM sodium phosphate buffer, pH 4.6). The tubes were thoroughly stirred on a vortex mixer and centrifuged for 10 min at 1,000 × *g*. Subsequently, 250 μl of the sample supernatants or extracting buffer solution for the blank was placed with 100 μl of azo-barley glucan substrate in 1.5-ml Eppendorf tubes, mixed and incubated for 30 min at 30°C. At the end of the incubation, 200 μl of a precipitating solution composed of 30% of a sodium acetate (10%)/zinc chloride (1%) buffer solution adjusted to pH 5 and 70% of a mixture of 95% ethanol with 5% methanol (industrial methylated spirits) were added to each tube. Finally, the tubes were centrifuged for 10 min at 1,000 × *g*, and the supernatants were placed in 96-well Bacti flat-bottom plates for measurement at 590 nm using an ELISA plate reader.

### Statistical Analysis

Data from all enzyme production were subjected to analysis of variance (ANOVA) as a completely randomized design using the general linear model procedure of SAS (35). Means were separated with Tukey's multiple-range test at *p* < 0.05 considered as significant.

Furthermore, to evaluate synergistic, antagonistic, or additive effects of the combinations of two or three strains of *Bacillus*, the data were subjected to a Bliss independence analysis considering single doses of the treatments with replicates (36). ANOVA analysis and Tukey's honestly significant difference test at 95% confidence were used to assess the difference between the predicted enzyme production and the measured enzyme production resulting from the combinations at the experimental level using STATGRAPHICS Centurion XV. If the difference between the predicted enzyme production and the experimental measurement of enzyme production in the combinations of *Bacillus* isolates was not significant (*p* ≥ 0.05), then the combination had an additive effect. However, the combination exhibited a synergistic effect when the experimentally measured enzyme production was more significant than the predicted enzyme production (*p* < 0.05). In contrast, when the experimentally measured enzyme production was significantly less than the predicted enzyme production (*p* < 0.05), then the combination had an antagonistic effect (37).

## Results

### *Bacillus* Genomes

A comparison with the results of the identification of *Bacillus* spp. isolates by 16S rDNA sequence analyses (7) and the whole-genome shotgun deposited at DDBJ/EMBL/GenBank of each *Bacillus* strains in Norum™ are summarized in Table 1. Sequencing of the three isolates generated 20,908,684, 14,341,220, and 17,436,246 reads for AM1002, AM0938, and JD17 isolates, respectively. The AM1002 genome assembly consisted of a 4,050,061-bp draft genome with 14 contigs more than 200 bp in length, with an average coverage of 694 ×, and an N50 value of 1,056,702, and GC content of 43.7%. The AM0938 genome assembly resulted in a 4,191,507-bp draft genome with 49 contigs more than 200 bp in length, with an average coverage of 470 ×, an N50 value 253,637, and GC content of 45.81%. Last, the JD17 genome assembly consisted of a 4,647,487-bp draft genome with 84 contigs more than 200 bp in length, with an average coverage of 579 ×, an N50 value of 626,821, and GC content of 45.58%. Only isolates AM1002 and AM0938 previously identified as *Bacillus subtilis* and *Bacillus amyloliquefaciens* by 16S rDNA sequence, respectively, were confirmed to be of the same genus and species. However, the whole-genome sequence of isolate JD17 gave *B. licheniformis* instead of *Bacillus amyloliquefaciens*. All genomes are deposited to NCBI and are available with accession numbers SAMN21479894, SAMN21479895, and SAMN21479896, respectively (Table 1).

**Table 1:** Identification of *Bacillus* spp. isolates by 16S rDNA sequence analyses (7) and whole-genome sequence by the National Center for Biotechnology Information (NCBI)^*^ present in the *Bacillus* direct-fed microbial (DFM) (Norum™).

**Isolate biosample**	**16S rDNA sequence analyses (closest match)**	**Whole-genome sequence Organism**	**NCBI accession number**
AM1002	*Bacillus subtilis* (100% ID)	*Bacillus subtilis* JAIWHY000000000	SAMN21479894
AM0938	*Bacillus amyloliquefaciens* (99.7% ID)	*Bacillus amyloliquefaciens* JAIWHX000000000	SAMN21479895
JD17	*Bacillus licheniformis* (99.6% ID)	*Bacillus licheniformis* JAIWHW000000000	SAMN21479896

* *This whole-genome shotgun project number PRJNA764204/SUB10393687 has been deposited at DDBJ/EMBL/GenBank*.

### Enzyme Determination

Table 2 shows the comparison of enzyme production of the three *Bacillus* strains and their possible combination when two or three strains were associated. Although the results in Table 2 show evident enzyme productive behaviors, it is impossible to determine synergistic, antagonistic, or additive effects in the groups consisting of combinations. Therefore, a Bliss independence analysis was performed to detect possible interaction effects between the different *Bacillus* strains.

**Table 2:** Evaluation of synergistic, additive, or antagonistic effects on the enzyme production behavior of three strains of *Bacillus* and their combinations by Bliss quantitative analysis.

**Treatments**	**Xylanase (mU/ml)**	**Cellulase (mU/ml)**	**Phytase (mU/ml)**	**Lipase (mU/ml)**	**Protease (U/ml)**	**β-Glucanase (mU/ml)**
1	27.54 ± 10.36^cd^	0.14 ± 0.02^d^	139.72 ± 4.64^ab^	0.87 ± 0.21^c^	100,550 ± 4,892.13^c^	150,186 ± 3,395.60^e^
2	112.97 ± 15.34^c^	0.54 ± 0.02^a^	167.28 ± 10.78^a^	0.92 ± 0.07^c^	188,491 ± 9,746.66^c^	525,676 ± 38,993.18^d^
3	2,472.83 ± 47.74^a^	0.34 ± 0.01^c^	144.53 ± 11.23^ab^	2.52 ± 0.10^b^	4,844,982 ± 375,063.1^b^	846,095 ± 40,691.79^b^
1–2	22.80 ± 8.59^d^	0.14 ± 0.01^d^	127.71 ± 14.45^b^	0.97 ± 0.07^c^	95,587 ± 3,221.85^c^	701,907 ± 22,311.42^c^
1–3	96.09 ± 12.55^cd^	0.47 ± 0.02^b^	131.31 ± 15.74^ab^	1.11 ± 0.12^c^	90,443 ± 2,324.68^c^	1,245,617 ± 62,679.77^a^
2–3	1,268.15 ± 48.04^b^	0.46 ± 0.03^b^	142.90 ± 14.82^ab^	2.93 ± 0.14^a^	13,399,146 ± 1,182,402^a^	961,245 ± 44,796.62^b^
1–2–3	27.58 ± 10.69^cd^	0.37 ± 0.02^c^	166.85 ± 7.68^a^	2.61 ± 0.14^ab^	94,269 ± 3,563.47^c^	949,230 ± 43,588.52^b^

*Data are expressed as mean ± standard error. N = 5. Values within columns with different lowercase superscripts differ significantly (p < 0.05)*.

#### Xylanase Production

**Figure 1:**
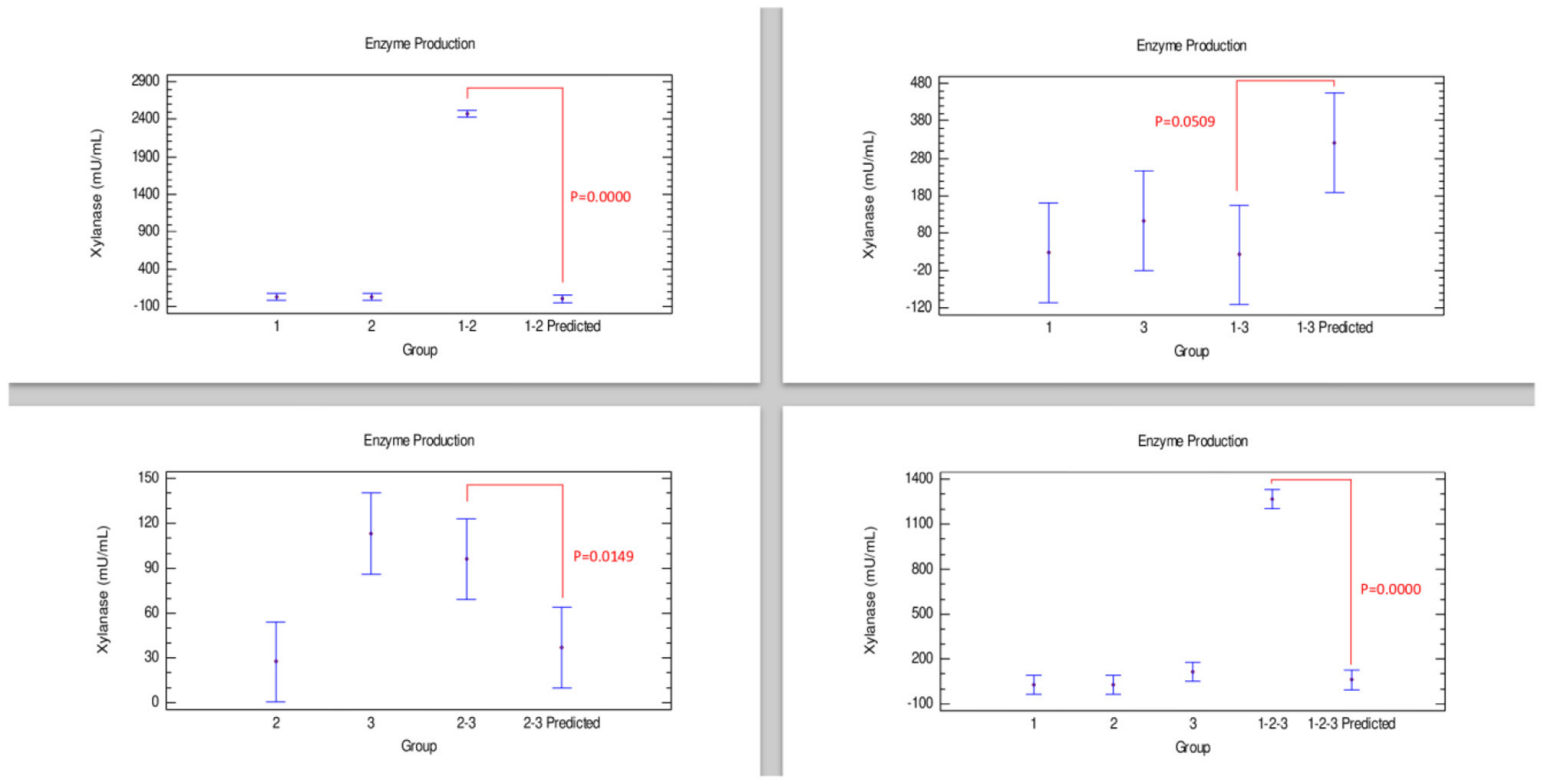
Comparison of groups to evaluate the interaction of three strains of *Bacillus* on the production of xylanase by Bliss independence analysis in single dose with replicates, considering the Tukey test at 95% confidence for comparisons. *p* < 0.05 indicates significant differences.

The results of xylanase production showed that *Bacillus* 3 had the highest production of this enzyme compared with the other strains, while combination 1–2 showed the lowest production. However, in Figure 1, a synergistic effect was observed in the combinations involving *Bacillus* strains 1–2 and 1–2–3, with the combination 1–2, producing the highest xylanase enzyme combinations. Meanwhile, the combination 2–3 showed an additive effect, with an enzyme production statistically similar to that of strain 2 individually. In contrast, the combination 1–3 presented statistically similar concentrations for each of the strains separately, indicative of an antagonistic effect.

#### Cellulase Production

**Figure 2:**
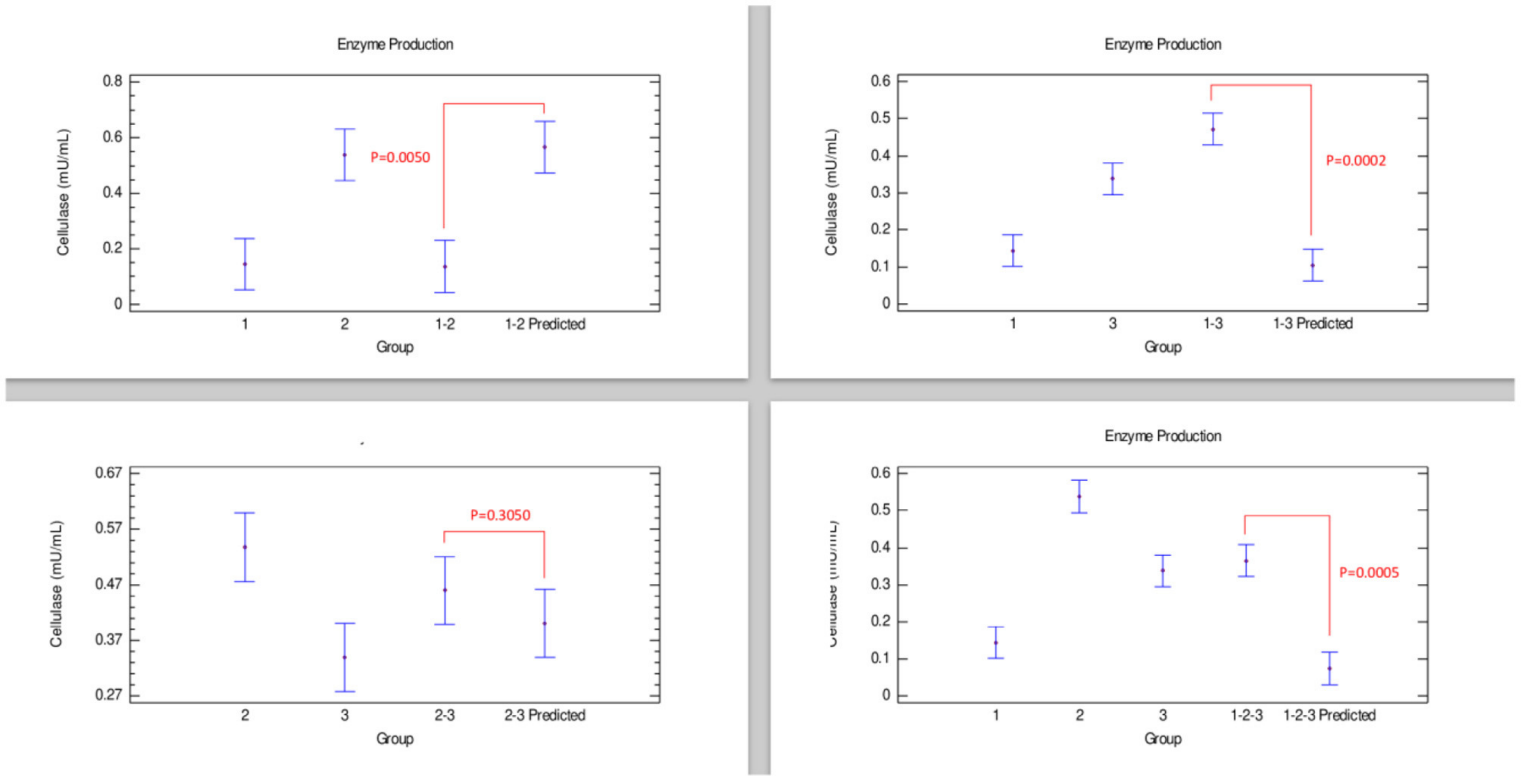
Comparison of groups to evaluate the interaction of three strains of *Bacillus* on the production of cellulase by Bliss independence analysis in single dose with replicates, considering the Tukey test at 95% confidence for comparisons. *p* < 0.05 indicates significant differences.

Cellulase production (Figure 2) showed that a combination of strains 1–3 had a synergistic effect in the production of this enzyme. Furthermore, combinations 2–3 presented an additive effect since the enzyme produced was statistically equal to both strains individually grown. Contrarily, when strains 1–2 and 1–2–3 were cultivated together, an antagonistic effect was observed in the cellulase production, with amounts of enzyme produced lower than those obtained with strain 2 individually grown (Table 2).

#### Phytase Production

**Figure 3:**
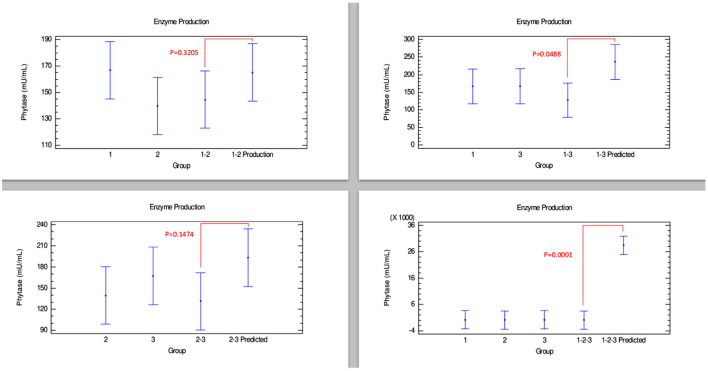
Comparison of groups to evaluate the interaction of three strains of *Bacillus* on the production of phytase by Bliss independence analysis in single dose with replicates, considering the Tukey test at 95% confidence for comparisons. *p* < 0.05 indicates significant differences.

Figure 3, corresponding to phytase production, shows no synergistic effects in any combination of the strains. Therefore, the joint growth of these strains did not provide any advantage to produce this enzyme. However, additive effects could have been observed in the combinations 1–2 and 2–3, since there were no differences between the enzyme amounts produced by the combination of strains and their predicted values. Furthermore, combinations 1–3 and 1–2–3 showed that phytase production could be affected when these strains are grown together, which can be interpreted as an antagonistic effect since there were statistically significant differences between the amounts produced by the combination of these strains and their predicted value.

#### Lipase Production

**Figure 4:**
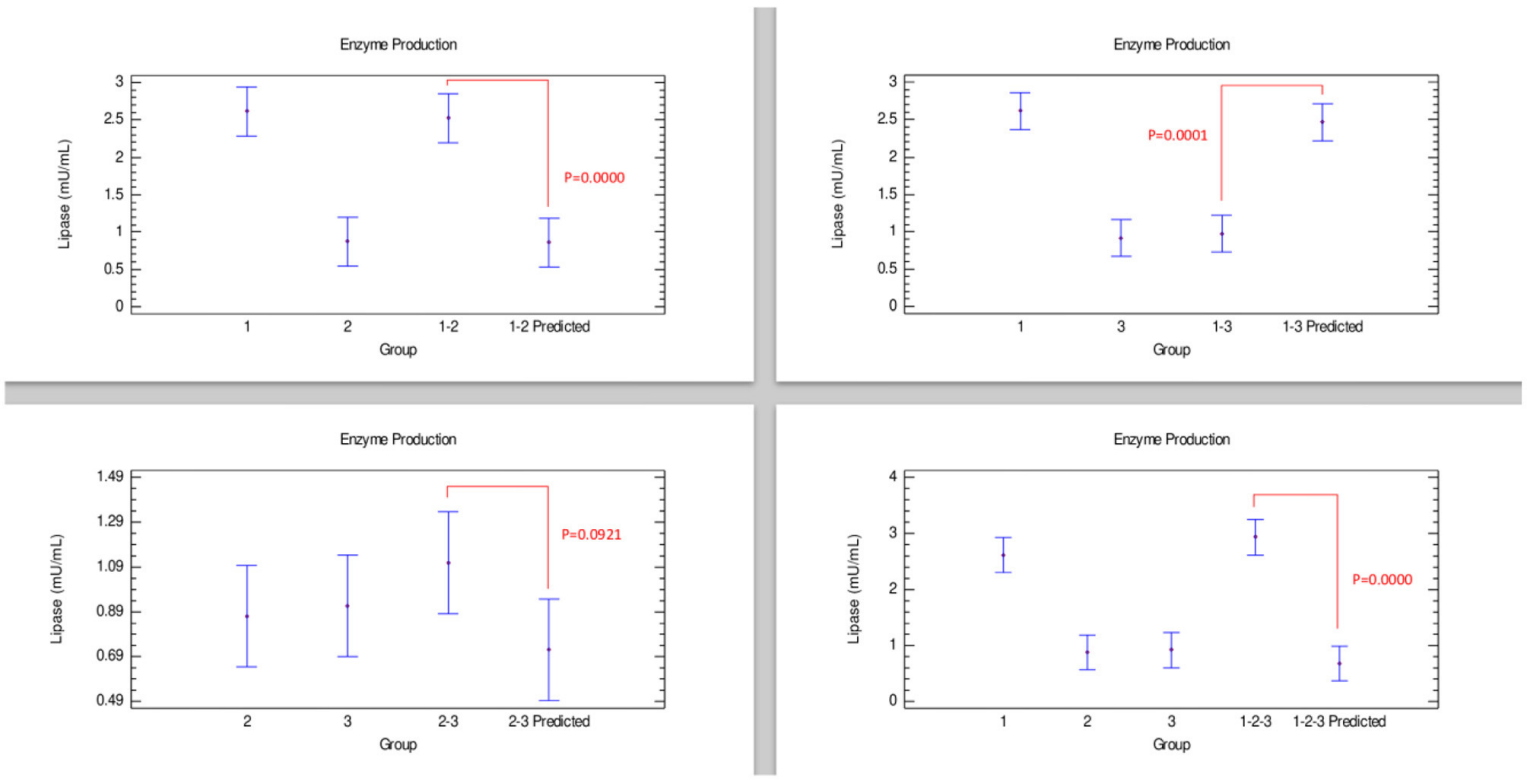
Comparison of groups to evaluate the interaction of three strains of *Bacillus* on the production of lipase by Bliss independence analysis in single dose with replicates, considering the Tukey test at 95% confidence for comparisons. *p* < 0.05 indicates significant differences.

According to the comparison of lipase production presented in Figure 4, combinations corresponding to the strains 1–2, and 1–2–3 exhibited an effect that could be categorized as additive since enzyme production was similar to individually grown strains. An additive effect was considered because the experimental enzyme production in the combinations was similar to *Bacillus* 1, although the predicted value was statistically lower than these groups. Considering the behavior of combinations 1–3, given that there were no significant differences between the experimental lipase production and its predicted value, it is suggested that an additive effect was presented. However, in the strain combination 1–3, a clear antagonistic effect between these strains could be observed.

#### Protease Production

**Figure 5:**
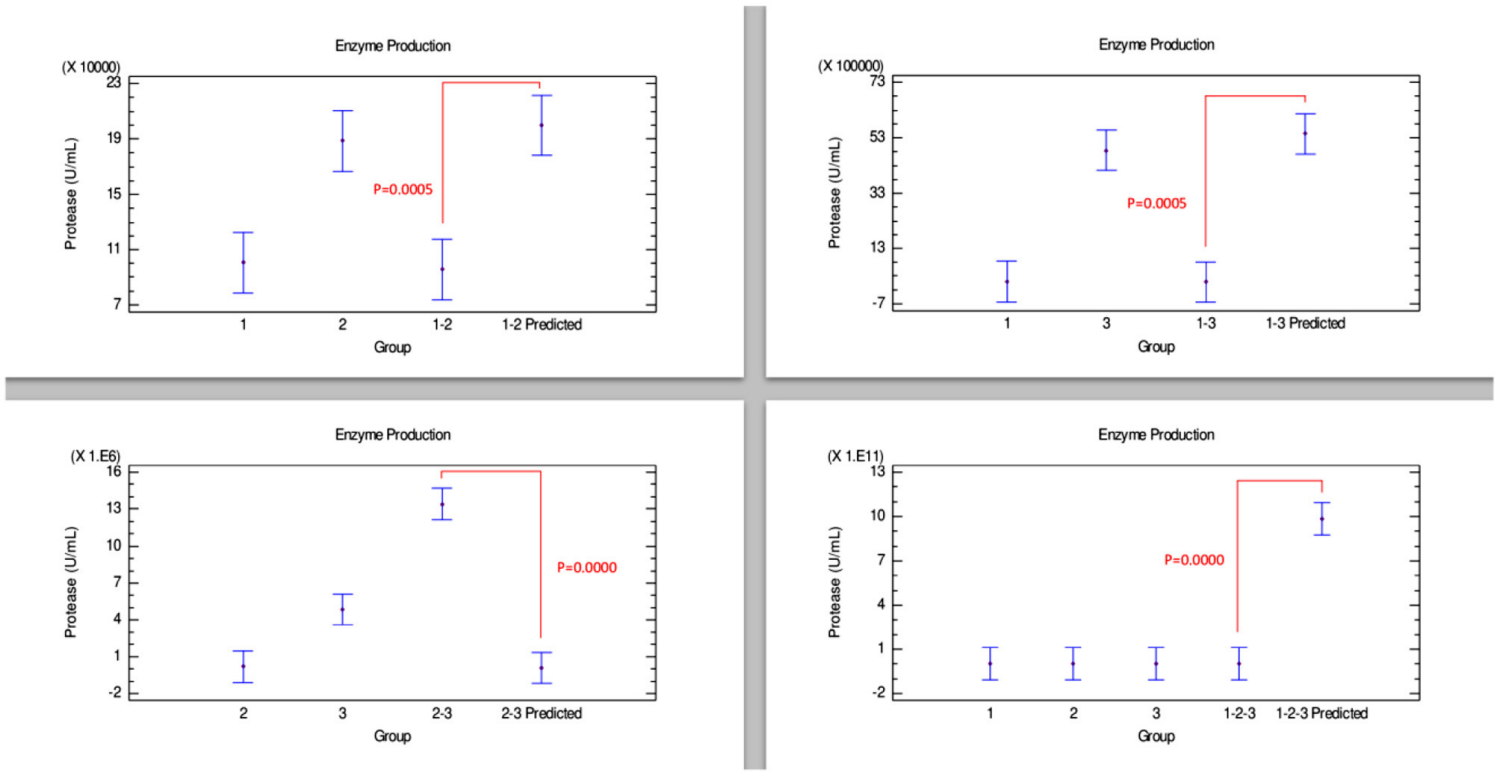
Comparison of groups to evaluate the interaction of three strains of *Bacillus* on the production of protease by Bliss independence analysis in single dose with replicates, considering the Tukey test at 95% confidence for comparisons. *p* < 0.05 indicates significant differences.

The results of the quantitative protease determination are summarized in Table 2. Interestingly, protease was the enzyme most produced by the *Bacillus* strains. However, in Figure 5, the combinations of strains 1–2, 1–3, and 1–2–3 presented an antagonistic effect since protease concentrations in the predicted group were significantly higher than the experimental combination group. Conversely, combination 2–3 showed an evident synergistic effect, being the combination that produced the highest amount of enzyme, even more than the amount produced by strains 2 and 3 individually.

#### β-Glucanase Production

**Figure 6:**
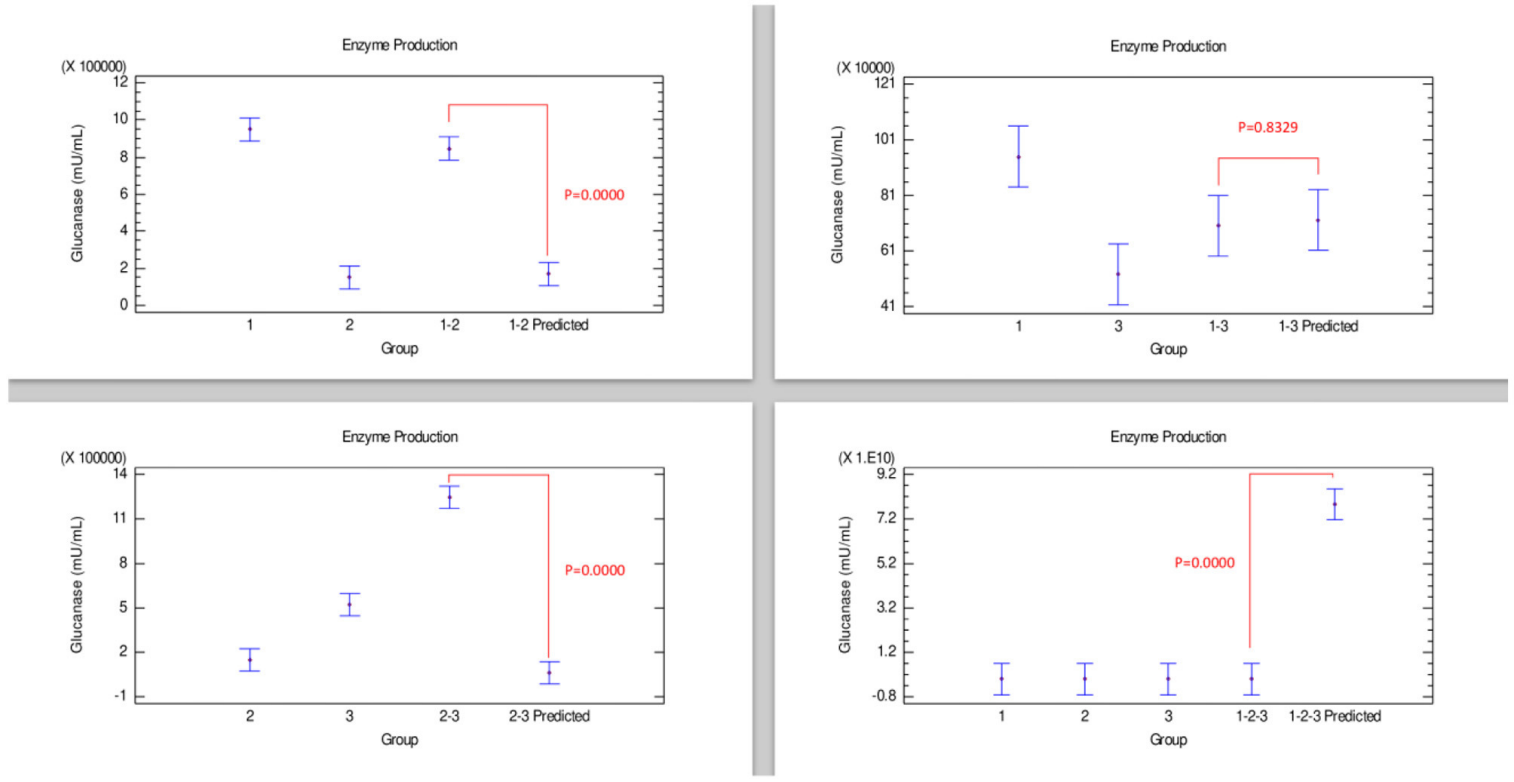
Comparison of groups to evaluate the interaction of three strains of *Bacillus* on the production of β-glucanase by Bliss independence analysis in single dose with replicates, considering the Tukey test at 95% confidence for comparisons. *p* < 0.05 indicates significant differences.

Figure 6 shows the results corresponding to β-glucanase production. The only combination, strains 2–3, showed a synergistic effect since this enzyme production was considerably improved compared with strains grown individually, which is corroborated with the statistical differences between the enzyme produced by the combination of these strains and their predicted values of the Bliss analysis. Conversely, a combination corresponding to the strains 1–2-−3 presented an effect that could be categorized as antagonistic since enzyme production was not improved compared when strains were grown individually, reflected in a notable statistical difference with the 1–2–3 predicted value. β-glucanase production by the combination of strains 1–2 and 1–3 appears to have an additive effect when grown individually because they did not show an improvement effect of β-glucanase production compared with only strain 1.

## Discussion

The recent increase in the use of *Bacillus* spp. strains as probiotics is due to bacterial resistance problems derived from the overuse of antibiotics at subtherapeutic doses, that is, growth-promoting antibiotics in production systems (38). This trend is marked global since several species of the genus *Bacillus* produce antimicrobial compounds, such as lipopeptides, surfactin, bacteriocins, and bacteriocin-like inhibitory substances, which affect both Gram-positive and Gram-negative harmful bacteria, making them the most promising viable alternatives to the use of antibiotics (8, 39). In addition, these probiotic bacteria can produce enzymes, such as amylase, protease, lipase, phytase, cellulase, β-glucanase, and xylanase, which have a beneficial effect not only on the digestibility and utilization of nutrients to promote the productive parameters (8) but also have an antimicrobial effect due to their ability to attack the pathogenic microorganism directly, interfere with biofilm formation, and/or catalyze reactions that result in the production of antimicrobial compounds (40, 41).

The current approach to probiotic strain selection is, in general, based on the experience of results gained in models with animals or the qualitative evaluation of these bacteria *in vitro*, ignoring the role of enzyme capacity (16). Therefore, in the present study, a quantitative analysis of the production of different enzymes of one *Bacillus subtilis* strain (1: AM1002) and two *Bacillus amyloliquefaciens* strains (2: AM0938 and 3: JD17), previously described (23) was performed, in order to know the effect of the inclusion of more than one strain within a product.

Analyzing the results of Table 2, the three *Bacillus* strains used in the present study produce different amounts of enzymes (xylanase, cellulase, phytase, lipase, protease, and β-glucanase). However, this production was modified when two or more *Bacillus* strains were combined, suggesting possible synergistic, antagonistic, or additive interactions. In this sense, to determine the possible effects produced in the combinations of the different *Bacillus* strains, a Bliss independence analysis of interactions was performed. This analysis is based on the probability theory for independent events and is used to analyze data for drug combinations by comparing observed vs. predicted responses (42, 43) and to identify synergistic, antagonistic, or additive effects (36, 37).

Considering the total results of the enzyme production present in each of the figures, the combination of *Bacillus* strains 2–3 was the one that presented the best results since their interaction led to synergistic and additive effects in all the cases. The assessment of effects by the Bliss independence analysis was important since each of the evaluated enzymes has different activity properties. In the case of xylanase, a common non-starch polysaccharide-degrading enzyme, it has shown an improvement in the digestibility of nutrients due to its effect on the decrease in intestinal viscosity, as well as on the improvement of immunity, reduction in the effects of *Salmonella* infections, and in the maintenance of intestinal epithelium in broilers challenged with *Clostridium perfringens* (44, 45).

Cellulase has important effects at the level of improvement of productive parameters and the increase in organic acids, such as propionic, which acts as a bacteriostatic reducing the colonization of pathogenic bacteria (46).

Phytase is included in poultry diets to improve the digestibility and assimilation of inorganic phosphorus, reducing environmental contamination by this element. Likewise, it has been shown to have a significant effect on the improvement of productive parameters and improvement in bone mineralization (47), in the same way as protease (48), the enzyme that was produced in higher concentration compared with the other enzymes in the present study.

Lipase has been shown to have a positive effect on the productive parameters in broilers and antimicrobial activity on Gram-negative bacteria since it acts at the level of lipopolysaccharides and exopolysaccharides present in broiler biofilms (49, 50).

Due to its direct effect on the decrease of intestinal viscosity and increase in the digestibility and use of fibers in poultry diets, as well as its antimicrobial activity against Gram-positive and Gram-negative bacteria, β-glucanase has been widely used in the poultry industry (33, 51).

## Conclusion

The present study showed that the selection of *Bacillus* strains based on quantitative methods of enzyme determination and adequate data analysis is essential to evaluate the effects that could occur in combinations of *Bacillus* strains to avoid interactions of the antagonistic type that could compromise the effectiveness of the treatments. The results demonstrated that the combination of *Bacillus* strains 2–3 was the group with the best prospects, which could lead to a new generation of DFMs with effects superior to those already evaluated in the combination of *Bacillus* strains 1–2–3, resulting in a viable alternative to the use of growth-promoting antibiotics. Further studies to confirm and expand the results in the present study using poultry models are currently being evaluated.

## Data Availability Statement

The datasets presented in this study can be found in online repositories. The names of the repository/repositories and accession number(s) can be found at: https://www.ncbi.nlm.nih.gov/genbank/, SAMN21479894, SAMN21479895, and SAMN21479896.

## Author Contributions

RD, OO, DH-P, BS-C, RM-G, JL, and GT-I conceptualized the study. PS, CA, DH-P, BS-C, MM, RM-G, JL, and RD handled the methodology. PS, CA, DH-P, BS-C, MM, and RM-G were in charge of the software. PS, CA, DH-P, BS-C, MM, RM-G, JL, and RD validated the study. PS, CA, DH-P, BS-C, and MM performed the formal analysis. PS, CA, DH-P, BS-C, MM, JL, and GT-I conducted the investigation. XH-V, PS, CA, DH-P, BS-C, and GT-I prepared and wrote the original draft. XH-V, PS, CA, DH-P, JL, BS-C, and GT-I contributed in the writing, review, and editing of the manuscript. PS, CA, DH-P, BS-C, BH, GT-I, OO, and RD were in charge of the project administration and funding acquisition. All authors contributed to the article and approved the submitted version.

## Funding

The authors declare that this study received funding from Eco-Bio/Euxxis Bioscience LLC and the United States Department of Agriculture and National Institute of Food and Agriculture, grant number #2019-69012-29905, as part of the Agriculture and Food Research Initiative—Sustainable Agricultural Systems. The funder was not involved in the study design, collection, analysis, interpretation of data, the writing of this article, or the decision to submit it for publication.

## Conflict of Interest

OO is employed by Biodigest S.A.S., and RD is employed by Nutriavícola S.A. The remaining authors declare that the research was conducted in the absence of any commercial or financial relationships that could be construed as a potential conflict of interest.

## Publisher's Note

All claims expressed in this article are solely those of the authors and do not necessarily represent those of their affiliated organizations, or those of the publisher, the editors and the reviewers. Any product that may be evaluated in this article, or claim that may be made by its manufacturer, is not guaranteed or endorsed by the publisher.
